# Neuroprotection Mediated through GluN2C-Containing N-methyl-D-aspartate (NMDA) Receptors Following Ischemia

**DOI:** 10.1038/srep37033

**Published:** 2016-11-15

**Authors:** Connie Chung, John D. Marson, Quan-Guang Zhang, Jimok Kim, Wei-Hua Wu, Darrell W. Brann, Bo-Shiun Chen

**Affiliations:** 1 Department of Neuroscience and Regenerative Medicine, Medical College of Georgia, Augusta University, Augusta, GA, 30912, USA; 2 Department of Neurology, Medical College of Georgia, Augusta University, Augusta, GA, 30912, USA

## Abstract

Post-ischemic activation of NMDA receptors (NMDARs) has been linked to NMDAR subunit-specific signaling that mediates pro-survival or pro-death activity. Although extensive studies have been performed to characterize the role of GluN2A and GluN2B following ischemia, there is less understanding regarding the regulation of GluN2C. Here, we show that GluN2C expression is increased in acute hippocampal slices in response to ischemia. Strikingly, GluN2C knockout mice, following global cerebral ischemia, exhibit greater neuronal death in the CA1 area of the hippocampus and reduced spatial working memory compared to wild-type mice. Moreover, we find that GluN2C-expressing hippocampal neurons show marked resistance to NMDA-induced toxicity and reduced calcium influx. Using both *in vivo* and *in vitro* experimental models of ischemia, we demonstrate a neuroprotective role of GluN2C, suggesting a mechanism by which GluN2C is upregulated to promote neuronal survival following ischemia. These results may provide insights into development of NMDAR subunit-specific therapeutic strategies to protect neurons from excitotoxicity.

NMDARs are ligand-gated ionotropic glutamate receptors that mediate excitatory synaptic transmission and are critical for synaptic plasticity and memory formation^1^,^2^. NMDARs also play a major role in the pathological events following excitotoxicity^3^. Overactivation of NMDARs, which causes excessive influx of calcium into the cell, is thought to be the primary step leading to neuronal excitotoxic damage. Functional NMDARs are tetramers consisting of two essential GluN1 subunits and two regulatory GluN2 (GluN2A-GluN2D) and/or GluN3 (GluN3A/GluN3B) subunits. NMDARs formed by different GluN1-GluN2 subunit combination exhibit distinct functional properties. Although NMDARs are broadly distributed throughout the brain, each GluN2 subunit displays unique spatiotemporal expression patterns. Among the GluN2 subunits, GluN2A and GluN2B are widely expressed. In contrast, the distribution of GluN2C is much more restricted. It is highly enriched in the cerebellum, thalamus and olfactory bulb^4^. GluN2C has also been found in other areas of the brain including the cortex and hippocampus in spite of being minimally detected^5^,^6^. Interestingly, increased expression of GluN2C is observed in the hippocampus following oxygen-glucose deprivation (an *in vitro* ischemia model)^7^,^8^. Consistently, GluN2C-immunoreactive positive cells have been found in the hippocampus in prolonged neonatal seizures^9^. These findings suggest that GluN2C may have an important role following stroke and seizure.

The GluN2C subunit confers unique properties on the NMDAR ion channels including low conductance openings and low sensitivity to magnesium^10^,^11^. This is true for both GluN1/GluN2C receptors expressed in heterologous cells and native NMDARs in the cerebellum. The disruption of the GluN2C gene eliminates the low conductance channels and reduces the decay time constant of NMDAR-mediated excitatory postsynaptic currents (EPSCs)^12^, reflecting a change in the composition of NMDARs. In addition, GluN2C-containing NMDARs require only modest depolarization to relieve Mg^2+^ blockade^5^. Because the voltage-dependent Mg^2+^ block of the NMDAR performs a crucial function in synaptic plasticity, this low sensitivity to Mg^2+^ implies that NMDARs with GluN2C can operate at a more negative membrane potential to regulate activity-induced synaptic changes. Moreover, recent studies have shown that GluN1/GluN2C receptors have an exceptionally low open probability^13^, which is approximately 44-fold and 10-fold less than the peak open probability of GluN1/GluN2A and GluN1/GluN2B receptors, respectively. Taken together, the unique channel properties and expression patterns indicate that GluN2C-containing NMDARs have a specific function in the brain.

We have previously shown that overexpression of GluN2C, in contrast to GluN2A and GluN2B, protects cerebellar granule neurons from excitotoxicity^14^. Although GluN2C has been shown to be upregulated in the hippocampus following ischemia, whether it has a neuroprotective role is not known. In this study, we confirm previous results, also showing that GluN2C is significantly increased following ischemia not only in mRNA but also protein levels. Using GluN2C knockout mice, we demonstrate that GluN2C promotes neuroprotective effects, preventing hippocampal neuronal damage following transient cerebral ischemia. We also show that overexpression of GluN2C protects hippocampal neurons from NMDA-induced excitotoxicity. Furthermore, the neuroprotective effect of GluN2C is likely due to a reduced calcium influx. Thus, our findings reveal a potential homeostatic mechanism that regulates NMDAR subunit composition in response to increased calcium influx. Through such a mechanism, not only the intracellular calcium level but also NMDAR signaling can be maintained at equilibrium.

## Results

### Cerebral ischemia induces GluN2C expression in the hippocampus

**Figure 1:**
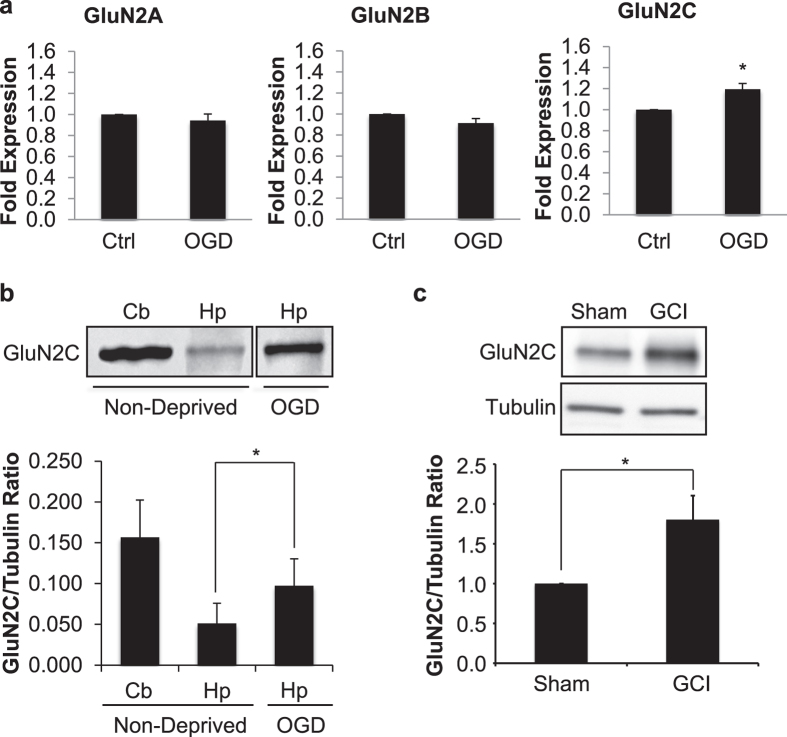
An increase in GluN2C expression, but not GluN2A or GluN2B, was observed following ischemia. (**a**) Summary data showing quantitative real time PCR results of GluN2A, GluN2B and GluN2C mRNA levels extracted from acute hippocampal slices following 4 min OGD and 90 min reperfusion. Ct-values are normalized to β-actin internal control. (**b**,**c**) Representative Western blot showing GluN2C upregulation in membrane fraction of acute hippocampal slices (labeled Hp in figure) following 4 min OGD and 3 hr reperfusion (**b**) or 12 min GCI and 6 hr reperfusion (**c**) (full-length blots are presented in Supplementary Fig. S8). Cerebellum lysate (labeled Cb in figure) was used as positive control for GluN2C expression. Graphs depict mean ± SEM (n = 3, *p < 0.05, Student’s t-test).

In an *in vitro* acute hippocampal slice model of cerebral ischemia, it has previously been shown that GluN2C mRNA levels increase following 4 minute (min) oxygen-glucose deprivation (OGD) in the hippocampus, whereas GluN2A and GluN2B levels remain stable and GluN2D is undetectable^7^,^8^. To confirm and extend previous studies, we measured both mRNA and protein levels of GluN2C under the same experimental conditions. We subjected acute hippocampal slices to 4 min OGD and 90 min reperfusion followed by quantification of mRNA levels of GluN2A, GluN2B and GluN2C using quantitative real-time PCR. Consistent with previous published reports, OGD significantly increased GluN2C mRNA levels (control, 1; OGD, 1.2 ± 0.1 fold expression; data were normalized to the corresponding control values; *p = 0.01; n = 3), whereas there were no significant changes in GluN2A and GluN2B mRNA levels (Fig. 1a). Next, we isolated the crude synaptosome (P2) fraction and assessed GluN2C expression at the protein level by Western blot analysis using a GluN2C specific monoclonal antibody (see Supplementary Fig. S1). Similar to the observed increase in GluN2C mRNA, we found a robust increase of GluN2C membrane protein in the hippocampus (non-deprived, 0.05 ± 0.02; OGD, 0.1 ± 0.03 GluN2C/tubulin ratio; *p = 0.03; n = 3) following 4 min OGD and 3 hr reperfusion compared to the non-deprived control group (Fig. 1b). The cerebellar lysate was used as a control, where it is known that GluN2C is enriched. No significant changes were observed in the expression of GluN2A and GluN2B (see Supplementary Fig. S2). To examine whether GluN2C expression is upregulated in the hippocampus *in vivo*, we induced global cerebral ischemia (GCI), which has previously been shown to induce neuronal degeneration in the hippocampus^15^, and measured GluN2C expression at 6 hours after GCI. We found that GluN2C expression was significantly increased (sham, 1; GCI, 1.8 ± 0.3 fold expression; data were normalized to the corresponding sham values; *p = 0.01; n = 3) following cerebral ischemia compared with sham controls (Fig. 1c). These results demonstrate that GluN2C can be induced in the hippocampus by a brief ischemic insult.

### The absence of GluN2C increases delayed neuronal cell death in the CA1 region following global cerebral ischemia in mice

**Figure 2:**
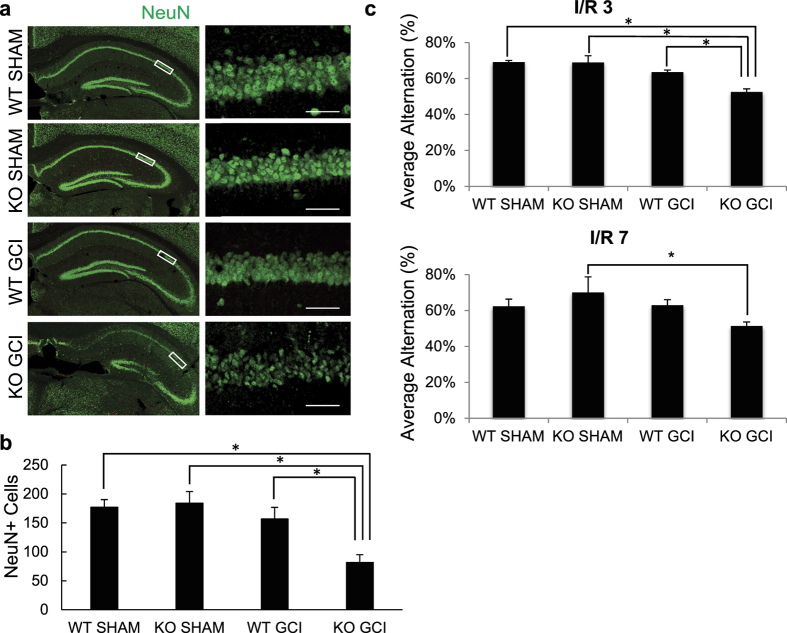
GluN2C^−/−^ mice display less CA1 surviving neurons and hippocampal-dependent functional loss following global cerebral ischemia (GCI). Both WT and KO (except sham groups) mice were induced with 15 min GCI. (**a**) Representative images of hippocampal sections comparing neuronal survival of WT sham, KO sham, WT GCI and KO GCI groups. Sections were stained with NeuN antibody (green). The left column depicts the hippocampus imaged using 10× objective and the right column images represent magnified CA1 areas (using 40× objective) highlighted by the white box. Cells that stained positively for NeuN staining were identified as surviving neurons. Scale bar, 50 μm. (**b**) Quantitiative summary of data showing the average number of NeuN+ cells (surviving neurons) per 320 μm length of medial CA1 region. (**c**) Average alternation percentage defined as “the number of alternating triads/(the total number of arm entries –2)” from Y-maze test at ischemia/reperfusion day 3 and 7 (n = 8 mice per GCI group, 3 mice per sham group, *p < 0.05, One-way ANOVA and Bonferroni test).

The functional role of GluN2C upregulation is unclear. To examine the role of GluN2C in the hippocampus *in vivo*, we used GluN2C^−/−^ knockout (KO) and GluN2C^+/+^ wildtype (WT) littermate mice to investigate whether the absence of GluN2C affects vulnerability to ischemic cell death following 15 min GCI and a 7 day reperfusion period. Neuronal survival was assessed by counting NeuN (a neuronal marker) positive cells within a 320 μm segment of the CA1 stratum pyramidale. Due to the severity of the ischemic injury sustained using longer than 20 min occlusion for both WT and GluN2C KO mice (see Supplementary Fig. S3), we chose to induce a shorter occlusion (15 min) that did not cause significant damage in WT CA1 hippocampal neurons, however was sufficient to induce a profound loss of NeuN staining in the hippocampal CA1 region in GluN2C KO mice compared with sham controls and WT GCI group (Fig. 2a). Quantification of NeuN-positive cells revealed that following GCI, GluN2C KO group had a significantly lower number of surviving neurons compared to WT sham, GluN2C KO sham, and WT GCI groups (Fig. 2b: WT sham, 177 ± 13; GluN2C KO sham, 184 ± 20; WT GCI, 157 ± 20; GluN2C KO GCI, 82 ± 13 NeuN positive cells, *p < 0.05, n = 3 for sham/8 for GCI groups). In addition, Fluoro-Jade C staining, a marker of neuronal degeneration, showed that GCI induced a marked Fluoro-Jade C-stained signal in GluN2C KO mice compared with sham controls and WT GCI group, suggesting that cerebral ischemia induced neuronal degeneration in the hippocampal CA1 region in GluN2C KO mice (see Supplementary Fig. S4). These findings demonstrate that GluN2C exerts a neuroprotective effect against cerebral ischemia *in vivo*, and in the absence of GluN2C expression, neurons become more vulnerable to ischemic insults.

### Effect of GluN2C on functional recovery following GCI

We assessed functional recovery after ischemia by administering behavior tests at ischemia/reperfusion (I/R) day 3 and 7. To assess cognitive impairment in spatial working memory, we administered the Y-maze spontaneous alternation test which has previously been validated as a hippocampal-dependent spatial task^16^. The alternation percentage was significantly reduced for GluN2C KO mice following GCI compared to WT sham, GluN2C KO sham and WT GCI groups at I/R day 3 (Fig. 2c: WT sham, 69.1% ± 1%; GluN2C KO sham, 69.0% ± 3.8%; WT GCI, 63.5% ± 1.2%; GluN2C KO GCI, 52.5% ± 1.7%, *p < 0.05, n = 3 for sham/8 for GCI groups). At I/R day 7, the alternation percentage for GluN2C KO mice following GCI was significantly reduced only when compared to GluNC KO sham group, whereas there was no significant difference comparing WT GCI and WT sham group (Fig. 2c: WT sham, 62.4% ± 4.0%; GluN2C KO sham, 70.0% ± 8.7%; WT GCI, 63% ± 3.1%; GluN2C KO GCI, 51.4% ± 2.2%, *p < 0.05, n = 3 for sham/8 for GCI groups). To evaluate gross locomotor activity, exploration habits, and anxiety, we used the open field test. There were no significant differences in overall distance traveled, mean speed, and time in center zone between WT and GluN2C KO groups for both sham and GCI conditions (see Supplementary Fig. S5a). Finally, the rotarod test determined no statistical difference in deficts of motor coordination and balance (see Supplementary Fig. S5b). Taken together, these results indicate a functional deficit in only hippocampal-dependent behavior testing in GluN2C KO mice following GCI, where the hippocampus is most susceptible to injury.

### Neuroprotection against NMDA-induced toxicity in GluN2C expressing hippocampal neurons

**Figure 3:**
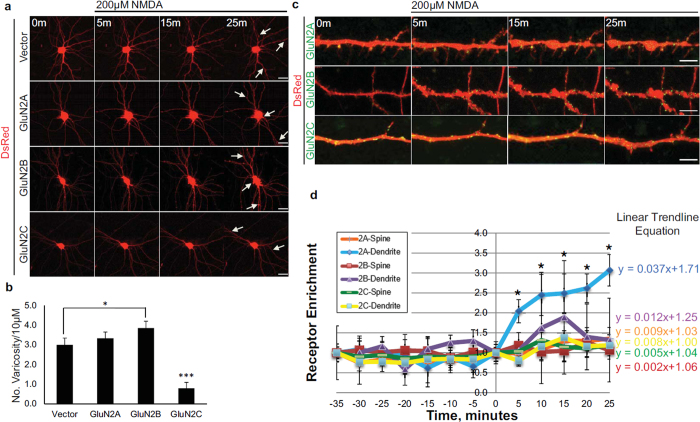
Overexpressed GluN2C mediates neuroprotection following NMDA-induced toxicity and surface expression levels remained unchanged in hippocampal neurons. (**a**) Time-lapse images of DIV 13 primary hippocampal neurons co-transfected with DsRed (to visualize cellular morphology) and SEP-tagged GluN2 constructs. Cells were exposed to 200 μM NMDA and imaged for 25 min. Arrows depict structural hallmarks of excitotoxic effects (dendritic varicosities and cell body swelling) in neurons expressing DsRed alone, GluN2A, or GluN2B. GluN2C-expressing neuron images show resistance to NMDA-induced toxicity. Scale bar, 10 μm. (**b**) Quantitiative summary of data showing the average number of dendritic varicosities per 10 μm length of dendrite (Vector, n = 18 dendritic segments, 6 cells; GluN2A, n = 18 dendritic segments, 6 cells; GluN2B, n = 21 dendritic segments, 7 cells; GluN2C, n = 24 dendritic segments, 8 cells, *p < 0.05, ***p < 0.01, One-way ANOVA and Bonferroni test). (**c**) Time-lapse images of zoomed in dendritic regions of hippocampal neurons co-expressing DsRed and SEP-tagged GluN2A, GluN2B, or GluN2C (green) before (0 min) and following administration of 200 μM NMDA. Scale bar, 2 μm. (**d**) Time course distribution of receptor enrichment for spines and dendritic segments from hippocampal neurons treated with NMDA (SEP-GluN2A, n = 24 spines, 6 dendritic segments, 4 cells; SEP-GluN2B, n = 31 spines, 7 dendritic segmens, 4 cells; SEP-GluN2C, n = 20 spines, 11 dendritic segments, 7 cells, *p < 0.05, One-way ANOVA and Bonferroni test).

Overexpression of GluN2C was previously shown to protect cerebellar granule neurons from NMDA-induced toxicity (a cell model that triggers excitotoxicity *in vitro*)^14^. We examined the effect of NMDA-induced toxicity in cultured primary hippocampal neurons co-expressing GFP-tagged GluN2A, GluN2B, GluN2C, or vector alone and DsRed (a red fluorescent protein that shows total distribution in dendrites and spines) to compare subunit-specific mediated changes in cellular morphology and to determine if GluN2C expression could indeed protect hippocampal neurons against excitotoxicity, as suggested by the *in vivo* results. Neurons were continuously exposed to 200 μM NMDA and time-lapse imaging was performed for 25 min. Cells expressing vector alone, GluN2A or GluN2B show morphological indicators of severe toxic effects, including dendritic beading or varicosities and cell body swelling (Fig. 3a). In contrast, expressing GluN2C is sufficient to mediate neuroprotective effects as cells were more resistant to injury, as evidenced by sustained normal cellular morphology. Quantification of dendritic varicosities revealed that following NMDA treatment, cells expressing GluN2C had significantly lower number of varicosities per 10 μm length of dendrite compared to vector, GluN2A-, and GluN2B-expressing cells. Cells expressing GluN2B had significantly higher number of varicosities compared to vector (Fig. 3b: Vector, 3.000 ± 0.343; GluN2A, 3.333 ± 0.313; GluN2B, 3.857 ± 0.344; GluN2C, 0.792 ± 0.294, *p < 0.05, ***p < 0.001, n = 6–8 cells). These results demonstrate that the neuroprotective role of GluN2C is neither cell-type specific nor confined to cerebellar granule cells but can also be evident when expressed in hippocampal neurons.

### NMDA receptor subunit-specific changes in surface expression following NMDA-induced toxicity

Due to the observed increase in GluN2C mRNA and protein following OGD, we next assessed whether this correlated with an increase in GluN2C surface expression. Therefore, we examined receptor subunit surface enrichment on spines and dendritic segments by co-expressing Super Ecliptic pHluorin (SEP)-tagged GluN2 subunits and DsRed in mature hippocampal neurons and performing time-lapse imaging during the course of NMDA treatment (Fig. 3c). SEP is a GFP variant that displays strong pH-dependent fluorescence and allows surface receptors to be dynamically visualized without antibody labeling^17^. Receptors on the surface membrane expose SEP to neutral environment and display fluorescence, whereas receptors in the low pH intracellular compartments are mostly nonfluorescent. This allowed us to assess surface expression of GluN2 subunits by live imaging. The experimental validity of the construct was tested by acid quenching experiments, where cells were incubated with standard HEPES solution (pH 7.4) and surface receptors became visible as a punctate distribution on dendritic areas. Cells were washed and incubated with MES acidic solution (pH 6.0) and the SEP signal became reversibly quenched. Upon reintroduction to standard HEPES solution, the SEP signal reappeared (see Supplementary Fig. S6). To compare the relative amounts of receptor on spines or dendritic segment for the different NMDAR subunits, we determined a surface enrichment value (volume normalized receptor fluorescence on spine or dendritic segment/volume normalized receptor fluorescence on adjacent dendritic segment), following the same quantification method as previously described^18^. GluN2A surface enrichment on dendritic segments significantly increased following 200 μM NMDA-induced toxicity at all time points tested relative to 0 min time point and GluN2B and GluN2C cell surface expression remained relatively stable over time (Fig. 3d). To compare the kinetic changes of receptor enrichment, we compared the curve-fitting linear trend line of each subunit and calculated the slope, which reflects the rate of change over time (Fig. 3d). The rate of change of receptor enrichment on dendritic segments for GluN2A, GluN2B and GluN2C following NMDA treatment was 0.037, 0.012 and 0.008, respectively. The rate of change on spines for GluN2A, GluN2B and GluN2C was 0.009, 0.002 and 0.005, respectively. We did not observe any significant changes in receptor enrichment for any NMDAR subunits under basal conditions when analyzed over 35 min without treatment. These results suggest that stable synaptic incorporation of SEP-GluN2C is sufficient to promote neuroprotective effects following NMDA-induced toxicity.

### GluN2C-expressing neurons display reduced intracellular Ca^2+^ influx following NMDA-induced toxicity

**Figure 4:**
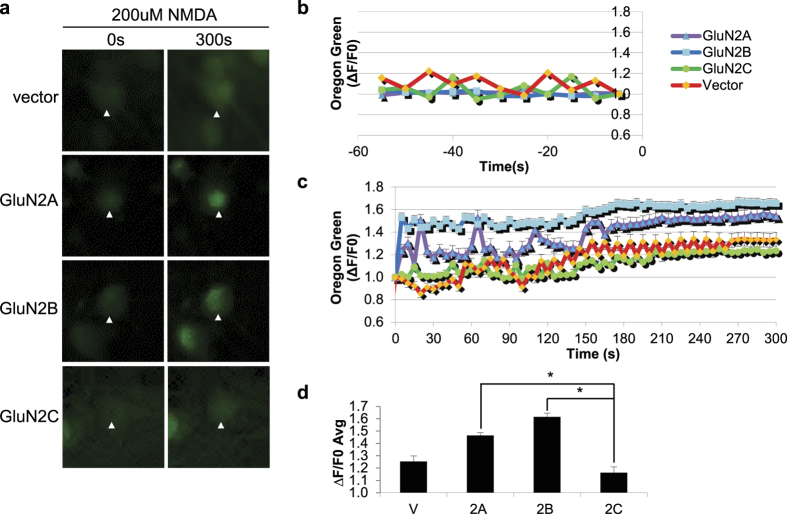
Reduced Ca^2+^ influx in GluN2C-expressing hippocampal neurons following NMDA treatment. (**a**) Time-lapse fluorescent images of hippocampal neuronal cell bodies co-transfected with DsRed (channel not shown for visual purposes) and WT-GluN2A, GluN2B, GluN2C, or vector alone. Oregon Green is depicted in green and its signal intensity directly correlates with the intracellular Ca^2+^ concentration. Images were obtained before (0 min), during (every 5 sec) and after (5 min) NMDA treatment. Only 0 min and 5 min time points are shown. (**b**) Time course distribution of basal level Ca^2+^ signaling 1 m before NMDA stimulation comparing vector, WT-GluN2A, WT-GluN2B, and WT-GluN2C transfected hippocampal neurons. (**c**) Time course distribution of Ca^2+^ responses evoked by NMDA treatment. (**d**) Mean ΔF/F0 of peak intensity time points (140 s–240 s) examined in (**c)**. (n = 3, *p < 0.05, Repeated Measures ANOVA and Bonferroni test).

It is well-established that the GluN2 subunit composition is critical for determining the channel properties of the receptor. In particular, GluN2C-containing NMDARs have distinct channel properties that set it apart from the other subunits, such as a low open probability, single-channel conductance and reduced Mg^2+^ sensitivity^1^,^19^,^20^. Dysregulation of NMDAR-mediated Ca^2+^ influx and subsequent intracellular Ca^2+^ overload is a critical link in the development of neuronal excitotoxicity following ischemia^21^. Therefore, we next investigated the possibility that GluN2C may functionally confer neuroprotection as a result of its unique channel properties and measured changes in intracellular Ca^2+^ concentration ([Ca^2+^]i). We co-expressed DsRed and GluN2A, GluN2B, GluN2C, or vector alone in hippocampal neurons and cells were loaded with the calcium indicator Oregon Green 488 BAPTA-1, AM and treated with 200 μM NMDA (Fig. 4a). The [Ca^2+^]i remained relatively stable and low during pre-NMDA stimulation (Fig. 4b), however NMDA treatment elicited a rapid rise in [Ca^2+^]i in GluN2A- and GluN2B-expressing cells which is sustained over the time course of 5 min (Fig. 4c). In contrast, GluN2C-expressing cells displayed significantly lower NMDA-induced changes in [Ca^2+^]i compared to GluN2A- and GluN2B-expressing neurons. The average kinetic changes in ΔF/F0 calculated for peak intensity time points (140 sec–240 sec) for vector-, GluN2A-, GluN2B-, and GluN2C-expressing cells was as follows: 1.25 ± 0.04, 1.46 ± 0.05, 1.61 ± 0.02, and 1.16 ± 0.03, respectively (Fig. 4d). The ΔF/F0 for GluN2C-expressing cells was significantly lower compared to GluN2A- and GluN2B-expressing cells. Furthermore, NMDA treatment exerted a dose-dependent effect in altering [Ca^2+^]i over a 5 min time course (see Supplementary Fig. S7). Importantly, at all NMDA doses tested (25 μM, 50 μM and 100 μM), GluN2C-expressing cells displayed the lowest average ΔF/F0 (1.03 ± 0.06, 1.18 ± 0.04, 1.10 ± 0.03, respectively). These findings strongly suggest that GluN2C mediates neuroprotection in the setting of NMDA-induced toxicity by modulating the Ca^2+^ permeability and minimizing receptor mediated [Ca^2+^]i overload in hippocampal neurons.

## Discussion

In the current study, we provide evidence that GluN2C is upregulated and plays a neuroprotective role following ischemia. First, we show that both mRNA and protein expression of GluN2C are increased in the hippocampus following ischemia. Second, mice lacking GluN2C are more vulnerable to neuronal damage induced by cerebral ischemia in the hippocampal CA1 region and these mice display cognitive impairment in spatial working memory. Third, cultured hippocampal neurons overexpressing GluN2C are protected from NMDA-induced toxicity. Finally, we show that GluN2C expressing neurons display reduced intracellular Ca^2+^ influx following NMDA-induced toxicity, which is consistent with the low channel open probabilities and reduced conductance of GluN2C-containing NMDARs. Thus, our findings suggest a homeostatic mechanism by which intracellular Ca^2+^ levels are maintained by upregulation of GluN2C to help prevent excessive Ca^2+^ entry following ischemia.

NMDAR overactivation has been shown to be necessary and sufficient to induce glutamate-mediated calcium toxicity in neurons^22^,^23^. Although functional NMDARs require both GluN1 and GluN2 subunits, the GluN2 subunit composition determines properties of the NMDAR channels as well as the coupling of NMDARs to distinct intracellular signaling pathways. Among the GluN2 subunits, GluN2A and GluN2B are predominantly expressed in the forebrain where stroke most frequently occurs. Interestingly, there is evidence that GluN2A and GluN2B have differential roles in mediating neuronal survival and death following ischemia^24^. GluN2B-containing NMDARs are linked to neuronal death signaling, whereas activation of GluN2A-containing NMDARs promotes neuronal survival. However, recent studies indicate that GluN2A can also promote neuronal death although less efficient than GluN2B under different experimental conditions^25^. Thus, subunit-specific rule in regulating neuronal survival or death signaling may be an oversimplification. While extensive efforts have focused on GluN2A and GluN2B subunits, GluN2C has been relatively neglected. We have previously shown that surface expression of GluN2C, unlike GluN2A and GluN2B, protects cerebellar granule neurons from NMDA-induced toxicity^14^. In this study, we show that GluN2C-deleted mice display greater sensitivity to neuronal damage in the CA1 region of the hippocampus following transient cerebral ischemia, strongly suggesting that GluN2C is neuroprotective.

In seeming contrast to our findings, previous studies by Kadotani *et al*. have shown that gene disruption of GluN2C attenuates focal cerebral ischemic injury after permanent occlusion of the middle cerebral artery (MCA)^26^, suggesting that GluN2C contributes to ischemic brain injury. Although GluN2C was reported to be expressed in the cerebral cortex in the previous studies, there is no change in GluN2C expression after permanent MCA occlusion. These findings contradict the observations that GluN2C is upregulated following *in vitro* ischemia by three groups including our current study^7^,^8^. The differences in the expression levels of GluN2C following ischemia may be due to different experimental conditions. One notable difference is that we induced transient ischemia and examined GluN2C expression after reperfusion, whereas Kadotani *et al*. used permanent occlusion without reperfusion. In addition, the GluN2C knockout mice were generated independently with different genetic background. Moreover, we chose the global cerebral ischemia model that is well known to cause selective neuronal injury in the CA1 region of the hippocampus^15^, where GluN2C expression has been shown to be upregulated. Importantly, our *in vivo* data, suggesting that GluN2C is neuroprotective, are supported by our *in vitro* results showing that hippocampal neurons expressing GluN2C are protected from NMDA-induced toxicity.

Although many selective NMDAR antagonists have been identified for the treatment of stroke, they all failed in clinical studies largely because of severe side effects caused by interfering the physiological functions of NMDARs^3^. Therefore, it is critical to develop novel therapeutics, which not only can reduce excessive Ca^2+^ influx but also maintain the physiological level of receptor function. The role of GluN2C in neuroprotection described here is two-fold. First, GluN2C-containing NMDARs exhibit an exceptionally low channel open probability^13^, which is in agreement with previous studies showing that deletion of GluN2C leads to higher charge transfer^12^,^27^. Consistently, we show that GluN2C expressing neurons has reduced Ca^2+^ influx following NMDA treatment. Second, GluN2C-containing NMDARs can trigger specific signaling pathways to activate downstream survival molecules. For example, activation of GluN2C-containing NMDARs has been shown to stimulate protein kinase B/Akt activity^14^, which plays a key role in neuronal survival pathways. How is GluN2C expression upregulated in response to ischemia? It has been shown that brain-derived neurotrophic factor (BDNF) mediates GluN2C upregulation in developing cerebellar granule neurons, which is controlled by neuronal depolarization^28^. During ischemia, neurons become depolarized. In addition, there is evidence that BDNF is increased in the hippocampus after the ischemic insult^29^. Thus, it is likely that the combination of these events results in GluN2C upregulation in the hippocampus after ischemia. A better understanding of exactly how GluN2C contributes to neuronal survival may lead to the development of new therapeutic tools for stroke and will be of interest to study in future work.

## Methods

### Animals

*In vivo* experiments were performed on male rats (Sprague Dawley) and male GluN2C^−/−^ (KO) and GluN2C^+/+^ (WT) littermate mice^30^ at 10–12 weeks of age. GluN2C^−/−^ mice (MMRRC, Stock #: 030658-UNC) were originally created on a mixed strain genetic background consisting of 129/SvJ, Swiss black, FVBN and C57BL/6. We crossed homozygous KO mice (GluN2C^−/−^) with C57BL/6J mice to obtain heterozygous GluN2C^+/−^ mice (backcrossed until ~97% C57BL/6J background), and then bred the GluN2C^+/−^ mice with each other to generate GluN2C^−/−^ and GluN2C^+/+^ mice. Prior to surgical procedures, animals were handled by the experimenter for 5 days (5 min per day) at the approximate time of the day behavioral tests were to occur. On the day of experiment, mice were placed in the test room >1 hr before tests. All procedures took place in the light phase of the light-dark cycle. Mice were genotyped using RED Extract-N-Amp Tissue PCR Kit (Sigma-Aldrich, St. Louis, MO). All animal experiments described in this study were carried out in accordance with the National Institutes of Health guide for the Care and Use of Laboratory Animals and were approved by the Institutional Animal Care and Use Committee of the Augusta University.

### Global cerebral ischemia (GCI)

GCI was performed as previously described^31^,^32^ with minor modifications. Anesthesia was induced with 2.0% isoflurane (Isothesia) driven by O_2_ flow, maintained during surgery with 1.5% isoflurane and reduced to 1.0% during occlusion using an inhalation mask. After midline cervical incision, both common carotid arteries (CCAs) of the mice were separated. A silk suture was placed around each artery and the vessels were occluded using micro vessel clips (World Precision Instruments) to induce 15 min ischemia in mice and 12 min ischemia in rats. The clips were then removed (right side first) and the blood flow through the arteries was confirmed before the wound was closed. Rectal temperature was maintained at 37 ± 0.5 °C throughout the experiment. After blood flow was restored, the mice were kept at 34 °C for 3 hr and recovered for 7 days. The animals of the sham group underwent identical procedures except that the CCAs were exposed and not occluded.

### Y-Maze

To assess cognitive deficits in short-term spatial working memory, the Y-Maze was performed as previously described^33^. The Y-maze (San Diego Instruments) consisted of three arms at 120° and was made of white plastic. Each arm was 7.5 cm wide and 38 cm long, and its three sides (except for the side adjoining the other arms) were surrounded by 12.5 cm high walls. Distal visual cues were placed around the Y-maze. A mouse was placed in the center of the maze and allowed to explore for 7 min. Behavior was monitored, recorded, and analyzed by the Any-Maze software. A mouse was considered to have entered an arm if the whole body (except for the tail) entered the arm and to have exited if the whole body (except for the tail) exited the arm. If an animal consecutively entered three different arms, it was counted as an alternating triad. Because the maximum number of triads is the total number of arm entries minus 2, the score of alternation was calculated as “the number of alternating triads/(the total number of arm entries − 2)”.

### Immunohistochemistry

Following the observation period, brains were fixed by cardiac perfusion with 4% PFA in PBS followed by 12 hr incubation in 4% PFA. Brains were then incubated for 24 hr in 20% sucrose in PBS, followed by 24 hr in 30% sucrose in PBS at 4 °C before freezing and sectioning on a Cryo-microtome yielding 30 μm coronal sections. Sections were then blocked in 5% BSA in PBS for 2 hr at room temperature (RT) and incubated in primary antibody overnight at 4 °C. NeuN (Millipore) antibodies were used at a 1:500 dilution in PBS containing 0.3% Triton X-100. Sections were then incubated in secondary antibodies conjugated to Alexa Fluor 488 (Life Technologies) for 2 hr at RT and mounted on glass slides with ProLong^®^ Gold Antifade mounting medium (Molecular Probes) for imaging analysis. Images were collected using a Zeiss LSM 700 confocal microscope using 10×, 40× and 63× objectives, and series of 0.05–1 μm optical sections were captured through the z-axis. For quantitative neuronal viability analysis, the number of NeuN-positive CA1 neurons per 320 μm length of the medial CA1 pyramidal cell layer was counted bilaterally in 5 sections per animal. The lower value between bilateral hemispheres was averaged across sections to provide the mean value.

### cDNA Constructs

Super Ecliptic pHluorin (SEP)-GluN2C construct was generated by cloning full-length rat GluN2C into the mammalian expression vector pRK5 and tagged with SEP between amino acids 36 and 37. SEP-GluN2A and SEP-GluN2B were kindly obtained from Roberto Malinow (UC San Diego). The SEP protein is in the extracellular N-terminus of the GluN2 subunit and exposed to an acidic intracellular vesicular environment until exocytosed into the plasma membrane. Once exposed to the neutral (7.4 pH) extracellular environment, there is an increase in fluorescence. All cDNA constructs used were verified by DNA sequencing.

### Primary Culture of Hippocampal Neurons and Transfection

Primary hippocampal cultures were prepared from embryonic day 18 Sprague Dawley rats as previously described^34^. Briefly, hippocampal tissue was dissociated with trypsin and plated on poly-D-lysine-coated coverslips at a density of 2 × 10^6^ cells/ml. The cells were cultured in Neurobasal medium supplemented with B27 and L-glutamine (all from Invitrogen). The cultures were maintained at 37 °C in 5% CO_2_. Cells were transfected at 13 days *in vitro* (DIV) using Lipofectamine 2000 (Invitrogen).

### Oxygen-Glucose Deprivation (OGD)

OGD was performed as previously described^7^,^8^ with minor modifications. Male 35–45 day old Sprague Dawley rats were anesthetized with isoflurane vapor and decapitated. Their brains were quickly (<1 min) removed and placed in 4 °C slicing saline solution consisting of (in mM): 108 NaCl, 2.5 KCl, 5 MgSO_4_, 45 NaHCO_3_, 12 Glucose, 0.5 Ascorbate, 0.5 CaCl_2_, 1 NaH_2_PO_4_ bubbled with 95% O_2_/5% CO_2_. The hippocampi were dissected and 400 μm transverse acute hippocampal slices were prepared using a vibrating slicer (Leica VT 1200S). Slices were placed onto floating mesh platforms in covered incubation chambers such that the slices were submerged in 34 °C (continuously bubbled with 95% O_2_/5% CO_2_) artificial cerebrospinal fluid (ACSF) consisting of (in mM): 127 NaCl, 2 KCl, 10 glucose, 1.2 KH_2_PO_4_, 26 NaH_2_CO_3_, 2 MgSO_4_, 2 CaCl_2_. After 90 min recovery, slices were subjected to either OGD (85% N_2_/5% CO_2_/10% H_2_ bubbled ACSF with 0 mM glucose and replaced with sucrose equimolarly) or non-deprived conditions for 4 min. Slices were then placed in normal oxygenated ACSF for 90 min (for mRNA analysis) or 3 hr (for protein analysis).

### RNA Extraction and Quantitative Real-Time PCR

RNAs from acute hippocampal slices were isolated using the TRI Reagent procedure (Invitrogen) according to manufacturer’s protocol. 2 μg of RNA was treated with RQ1 RNase-Free DNase and RNase inhibitor (Promega), and the purity and concentration was verified with NanoDrop 2000 Spectrophotomer (Thermo Scientific). Single-stranded cDNA was synthesized from extracted RNA with SuperScript III First-Strand Synthesis System (Invitrogen). The SsoAdvanced SYBR Green Supermix Reagent (Bio-Rad) and CFX96 Touch^TM^ Real-Time PCR Detection System (Bio-Rad) were subsequently used and samples were analyzed for the expression of 4 target genes of interest: GluN2A (forward primer 5′-AGGACAGCAAGAGGAGCAAG-3′, reverse primer 5′-ACCTCAAGGATGACCGAAGA-3′, product size: 174 bp), GluN2B (forward primer 5′-TGAGTGAGGGAAGAGAGAGAGG-3′, reverse primer 5′-ATGGAAACAGGAATGGTGGA-3′, product size: 249 bp), GluN2C (forward primer 5′-GGGCTCCTCTGGCTTCTATT-3′, reverse primer 5′-GACAACAGGACAGGGACACA-3′, product size: 162 bp) and β-actin used as a loading control (forward primer 5′-TGACAGGATGCAGAAGGAGA-3′, reverse primer 5′-TAGAGCCACCAATCCACACA-3′, product size: 104 bp). Each experiment included a template-free control. A standard curve for each gene was generated by serial dilutions of a standard. The PCR products were analyzed by the DNA melting curve. The experiment was repeated three times and the relative quantities of PCR products were estimated with respect to the amount of β-actin product using the ΔCt method: (2Ct of β-actin − Ct of Target Gene) × 100.

### Immunoprecipitation and Western Blot

The crude synaptosome (P2) fraction from acute hippocampal slices was prepared as described previously^35^. The P2 fraction was immunoprecipitated with rabbit GluN2C antibodies (Chemicon) overnight at 4 °C and then incubated with Protein A magnetic beads (Dynabeads) and 1 μg/ml BSA for 1 hr at 4 °C and washed with 1% Triton X-100 in PBS containing protease inhibitor cocktail (Pierce). Immunoprecipitates were resolved by SDS-PAGE and immunoblotted with mouse GluN2C antibodies (NeuroMab). Antibody specificity was tested using mouse cerebellum lysate from GluN2C^−/−^ and GluN2C^+/+^ (see Supplementary Fig. S1). The experiment was repeated three times and quantified using ImageQuant software.

### *In Vitro* Neurotoxicity Assay

Hippocampal neurons were subjected to neurotoxicity as previously described^14^. At DIV13, primary hippocampal cells cultured in glass chamber slides (Lab-Tek) were transfected with DsRed and pRK5, SEP-GluN2A, SEP-GluN2B, or SEP-GluN2C. At DIV15, the medium was replaced with standard HEPES-buffered saline solution (7.4 pH) containing (in mM): 116 NaCl, 5.4 KCl, 0.80 MgSO_4_, 1.01 NaH_2_PO_4_, 25 NaHCO_3_, 12 HEPES, 5.5 D-glucose, 1.8 CaCl_2_. Time-lapse imaging was performed for 25 min following 25–200 μM NMDA (Tocris Bioscience) treatment and images collected every 2.5 min. Confocal images were acquired using either 40× or 63× objective. Serial optical sections were obtained at 0.5–1 μm intervals. Neurotoxicity was determined by cell morphology (cell body swelling and dendritic varicosities) visualized by DsRed fluorescence. Quantification of dendritic varicosities was performed on three dendrites at least 10 μm away from the soma that were randomly chosen, and the number of beaded structures along the same 10 μm length of dendrite was counted. The data were expressed as the number of varicosities per 10 μm of dendrite. Receptor spine/dendritic segment enrichment was determined as previously described^18^. Enrichment of receptors on spines is defined as: (spine green/spine red fluorescence)/(dendrite green/dendrite red fluorescence).

### Calcium Imaging

Oregon Green 488 BAPTA-1, AM (Molecular Probes by Life Technologies) was used in accordance with the manufacturer’s protocol. Briefly, a 10 μM working solution was freshly prepared on the day of the experiment and applied to the cells for 30 min at room temperature. The cells were subsequently washed three times with ACSF and time-lapse imaging was performed for 1 min pre-NMDA stimulation and 5 min post-NMDA stimulation and images collected every 5 sec. The integrated intensity of Oregon Green signal within the defined region of interest (cell somata) was normalized to DsRed and each frame was analyzed using the Time Series Analyzer plugin in ImageJ. ΔF/F0 is defined as the normalized change in fluorescence intensity relative to the resting fluorescence intensity at 0 sec.

### Data Analysis and Statistics

Results from multiple repeats are expressed as average ± SEM and analyzed using Student’s t-test, Repeated Measures, or One-Way ANOVA, as appropriate. Following ANOVA, post hoc Bonferroni procedure was used to determine whether the data are statistically different from each other. Differences among groups were considered significant if P < 0.05.

## Additional Information

**How to cite this article**: Chung, C. *et al*. Neuroprotection Mediated through GluN2C-Containing N-methyl-D-aspartate (NMDA) Receptors Following Ischemia. *Sci. Rep.* **6**, 37033; doi: 10.1038/srep37033 (2016).

**Publisher’s note**: Springer Nature remains neutral with regard to jurisdictional claims in published maps and institutional affiliations.

## Supplementary Material

Supplementary Information
