# Supplementary material for: Neuroprotection Mediated through GluN2C-Containing N-methyl-D-aspartate (NMDA) Receptors Following Ischemia
Source: Sci Rep. 2016 Nov 15;6:37033. doi: 10.1038/srep37033 (PMC5109474; doi:10.1038/srep37033)
Supplement: Supplementary Information [file srep37033-s1.doc]

**Supplementary Information**

**Neuroprotection Mediated through GluN2C-Containing N-methyl-D-aspartate** **(NMDA)** **Receptors Following Ischemia**

Connie Chung^1,2^, John D. Marson^1,2^, Quan-Guang Zhang^1,2^, Jimok Kim^1,2^, Wei-Hua Wu^1,2^, Darrell W. Brann^1,2^ and Bo-Shiun Chen^1,2,3^

^1^Department of Neuroscience and Regenerative Medicine and ^2^Department of Neurology, Medical College of Georgia, Augusta University, Augusta, GA, 30912, USA

^3^To whom correspondence should be addressed: Bo-Shiun Chen, Department of Neuroscience and Regenerative Medicine and Department of Neurology, Medical College of Georgia, Augusta University, CA-3008, 1120 15^th^ Street, Augusta, GA 30912, Tel. 706-721-5926; Fax 706-721-8752; email: [bochen@augusta.edu](mailto:bochen@augusta.edu)


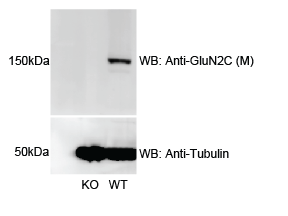


**Supplementary Figure S1.** Western blot depicting specificity of monoclonal NeuroMab anti-GluN2C antibody used for experiment represented in Figure 1. Crude membrane fraction of GluN2C KO and WT cerebellum lysate was used to detect endogenous GluN2C protein. Bottom panel depicts α-tubulin used as loading control.


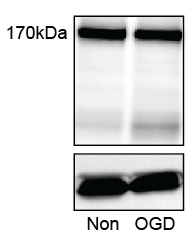


WB: Anti-GluN2A/GluN2B

WB: Anti-Tubulin

**
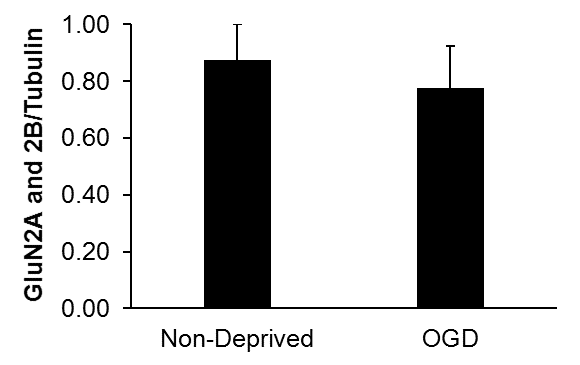
**

**Supplementary Figure S2.** Representative Western blot showing no significant change in GluN2A/GluN2B following 4 min OGD and 3 hr reperfusion. The NMDAR antibody recognized both GluN2A and GluN2B, which have similar molecular weights. Bottom panel depicts α-tubulin used as loading control. Graphs depict mean ± SEM (n = 3, *p < 0.05, Student’s t-test).


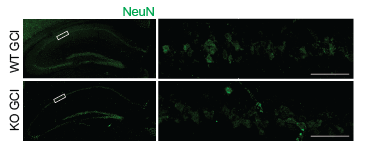


**
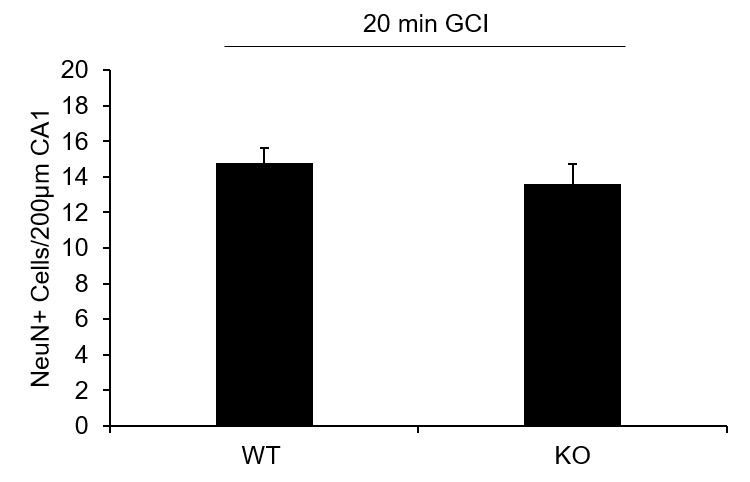
**

**Supplementary Figure S3.** Both GluN2C-/- (KO) and WT mice display similar severe CA1 neuronal cell death following 20 minutes of global cerebral ischemia (GCI). Representative images of hippocampal sections comparing neuronal survival of WT GCI and KO GCI groups. Sections were stained with NeuN antibody (green). The left column depicts the hippocampus imaged using 10× objective and the right column images represent magnified CA1 areas (using 40× objective) highlighted by the white box. Cells that stained positively for NeuN staining were identified as surviving neurons. Scale bar, 50 µm. Quantitatve analysis of NeuN+ cells within 200μm CA1 region demonstrates no significant difference between WT and KO groups following 20 minutes of GCI. Graph depicts mean ± s.d. (n=3, *p < 0.05, Student’s t-test).


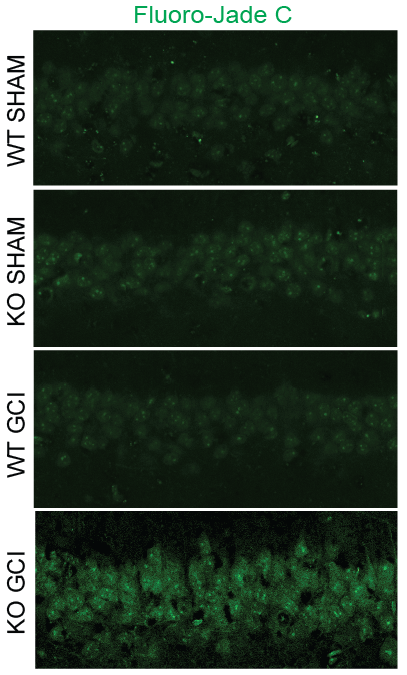

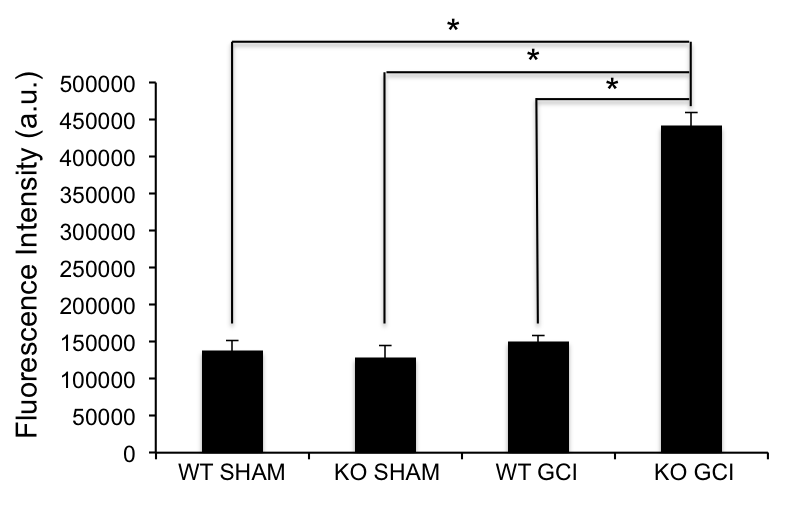


**Supplementary Figure S4.** GluN2C^-/-^ mice display greater CA1 neuronal cell death following GCI. Both WT and GluN2C KO (except sham groups) mice were induced with 15 min GCI. Hippocampal sections were stained with Fluoro-Jade C (green), a marker of neuronal degeneration. Cells that stained negatively for Fluoro-Jade C staining were identified as surviving neurons. The images shown are representative of hippocampal sections of WT sham, GluN2C KO sham, WT GCI and GluN2C KO GCI groups. Graph depicts mean fluorescence intensity of Fluoro-Jade C staining ± s.d. (n=3, *p < 0.05, ANOVA and Bonferroni test). Scale bar, 50 μm

**
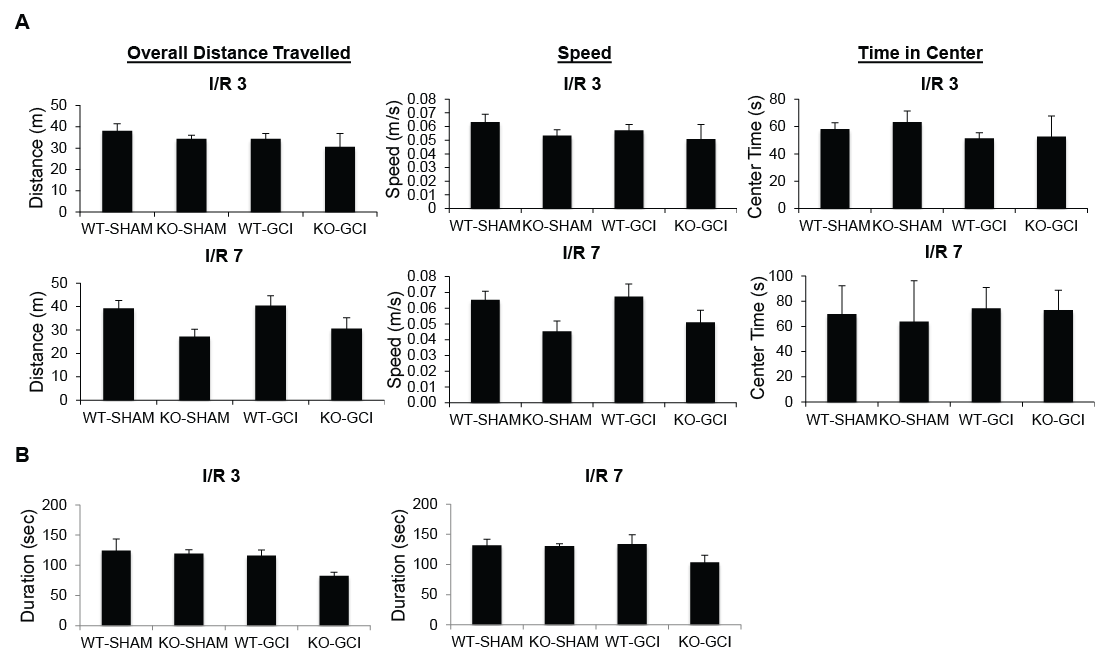
**

**Supplementary Figure S5.** No impairment observed in open field test (gross motor activity, exploratory behavior and anxiety assessment) and rotarod test (to assess motor coordination and balance) following GCI for WT and GluN2C KO groups. A, Summary of data from open field test comparing performance over 10 min observation period between WT and KO (sham and GCI groups) in 3 different measures: overall distance traveled, mean speed, and time in center zone. The area of 20 × 20 cm^2^ in the middle of the arena was set as a center area. The tendency of a mouse to avoid this center area was used as an indication of anxiety level. No significant differences were found between groups at I/R day 3 and when tested again at I/R day 7. B, Summary of data from rotarod test showing latency to fall. Mice were placed on an accelerating rotarod (4 rpm to 40 rpm) and latency to fall was recorded. No significant differences were found at both I/R day 3 and 7 (n = 8 mice per GCI group, 3 mice per sham group, *p < 0.05, One-way ANOVA and Bonferroni test).

**
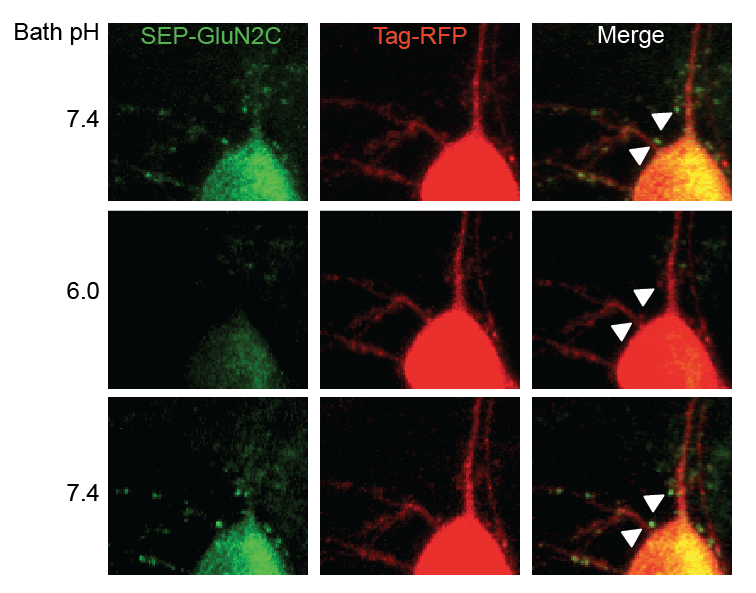
**

**Supplementary Figure S6.** Super ecliptic pHluorin (SEP)-tagged GluN2C allows visualization of surface receptors. Dissociated neuronal cultures were co-transfected at DIV 7 with Tag-RFP (red) and SEP-tagged GluN2C (green). One week after transfection, cells were incubated with standard HEPES solution (pH 7.4, top row) and imaged live. Cells were then washed and incubated with MES acidic solution (pH 6.0) and imaged again (middle row). The SEP signal becomes reversibly quenched by the change in pH. Cells were returned to the standard HEPES solution (bottom row). Arrowheads depict areas where SEP-GluN2C becomes visible under neutral pH conditions and quenched under acidic conditions.

**
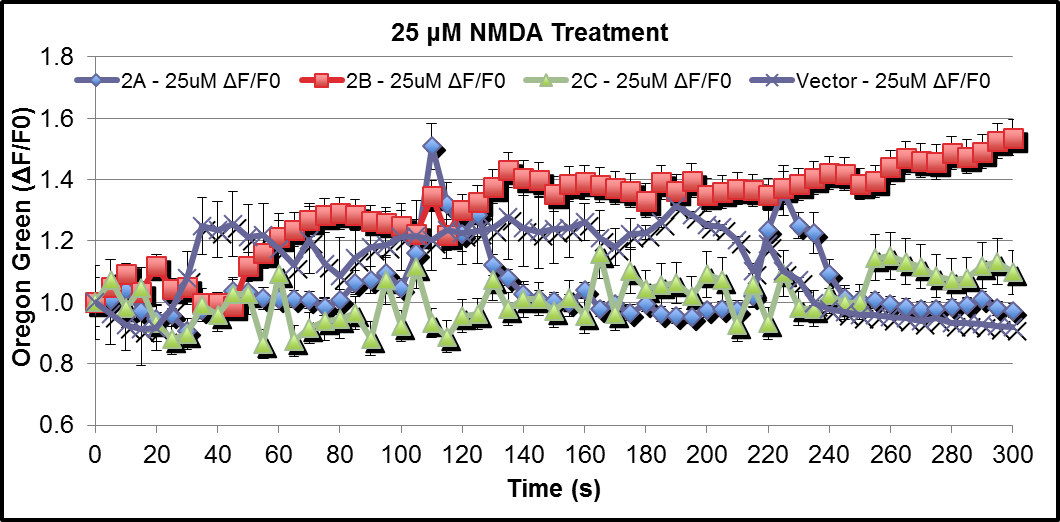

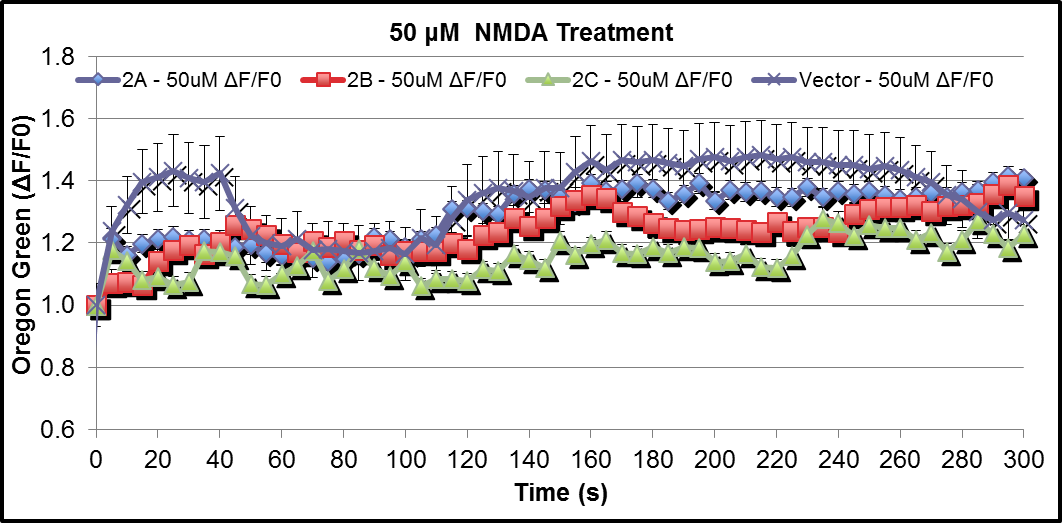

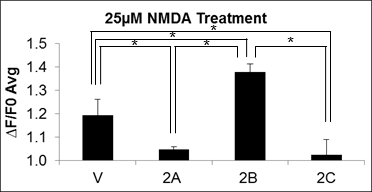
**

**
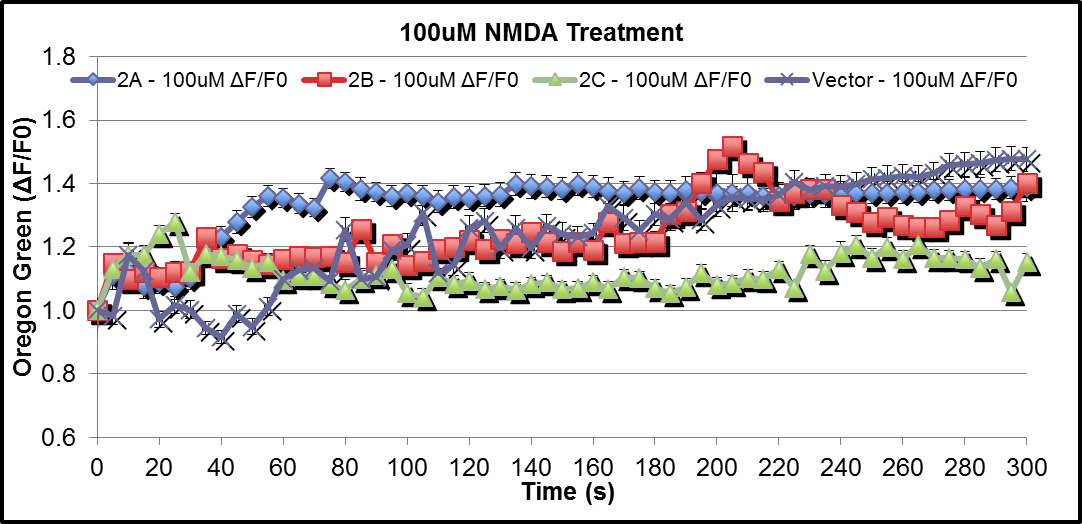

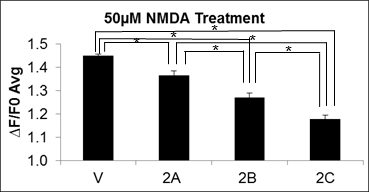

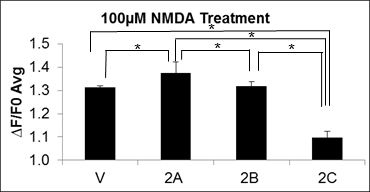
**

**Supplementary Figure S7.** Left column graphs display time course distribution (0 sec – 300 sec) of Ca^2+^ responses evoked by 25, 50, and 100 μM NMDA treatment. Right column graphs display mean ΔF/F0 of peak intensity time points (140 sec -240 sec). (n = 3, *p < 0.05, Repeated measures ANOVA and Bonferroni test).

**
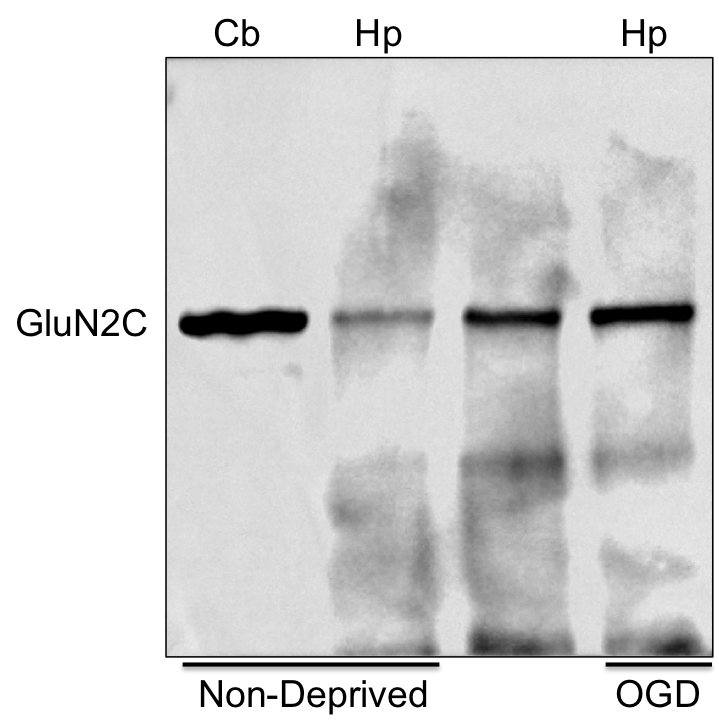
Fig.1b**

*


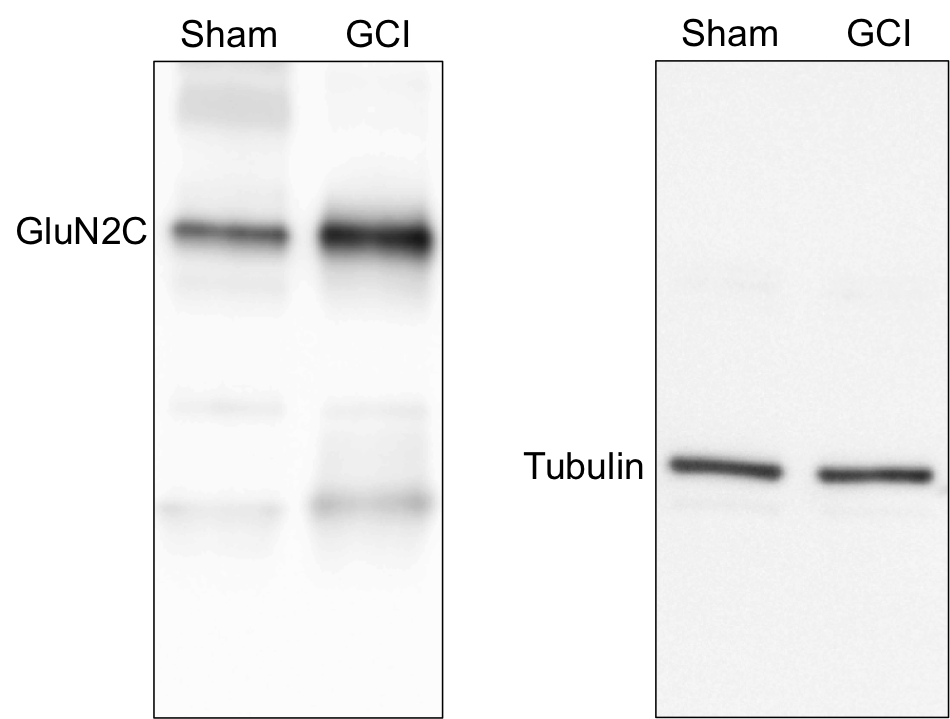
**Fig.1c**

**Supplementary Figure S8. Full-length blots of figure 1b and 1c.** Representative Western blot showing GluN2C upregulation in membrane fraction of acute hippocampal slices following 4 min OGD and 3 hr reperfusion (b) or 12 min GCI and 6 hr reperfusion (c). Cerebellum lysate was used as positive control for GluN2C expression. * In figure 1b lane 3, hippocampal slices were subjected to 30 min GCI and 3 hr reperfusion, which was not shown in the main figure.

**Supplementary methods**

*Immnoblotting.* Antibody specificity was tested using the crude synaptosome (P2) fraction of mouse cerebellum lysate from GluN2C^-/-^ and GluN2C^+/+^. Immunoprecipitates were resolved by 6% SDS-PAGE and immunoblotted with mouse monoclonal GluN2C antibodies (NeuroMab) at a 1:1000 dilution. The experiment was repeated three times and quantified using ImageQuant software. A ratio of GluN2C protein to α-tubulin loading control was determined.

*Fluoro-Jade C Staining.* Hippocampal sections were washed in PBS containing 0.1% Triton X-100 two times for 10 min each and then incubated for 20 min with fluorescent Fluoro-Jade C dye (Millipore) diluted in PBS as recommended by the manufacturer (Millipore). Staining solution was removed and sections were washed with PBS-Trition X-100 three times for 15 min each, then PBS one time for 15 min and mounted on glass slides with ProLong Gold Antifade mounting medium (Molecular Probes) for imaging analysis.

*Open Field Test.* An open field test was used to detect any gross motor deficits, anxiety-like behavior, and exploratory behavior (Prut & Belzung, 2003). Mice were placed in the center of a chamber [40 cm × 40 cm × 30 cm (L × W × H)] made of wood coated with black plastic under the illumination of 100 lux. The floor was covered with a sawdust bedding material. After each trial, the sawdust was redispersed to remove odor trails. Each trial was recorded by a webcam (Logitech C920) and the Any-Maze software (Stoelting). The area of 20 × 20 cm^2^ in the middle of the arena was set as a center area in the analysis software. The tendency of a mouse to avoid this center area was used as an indication of anxiety level. During a 10 min observation period, distance traveled, mean speed, time in center zone, and distance traveled in center zone were recorded and analyzed.

*Rotarod Test.* To evaluate sensorimotor coordination, mice were placed on an accelerating rotarod (Rotor-Rod System; San Diego Instruments) and assessed for ability to maintain balanced on the rotating bar that accelerated from 4 to 40 rpm over a 2 min period. Prior to testing, mice were trained for 3 consecutive days. Mouse latency to fall from the rod was recorded and mean time spent on the rotarod over a period of 3 successive trials was scored.

*Acid Quenching in Dissociated Neurons*. Super Ecliptic pHluorin (SEP)-GluN2C construct was generated by cloning full-length rat GluN2C into the mammalian expression vector pRK5 and tagged with SEP between amino acids 36 and 37 in the extracellular N-terminus. The SEP protein is exposed to an acidic intracellular vesicular environment until exocytosed into the plasma membrane. Once exposed to the neutral (7.4 pH) extracellular environment, there is an increase in fluorescence. The experimental validity of the construct was tested by acid quenching experiments. Dissociated neuronal cultures were co-transfected at DIV 7 with Tag-RFP and SEP-tagged GluN2C. One week after transfection, cells were incubated with standard HEPES solution (pH 7.4) containing (in mM): 116 NaCl, 5.4 KCl, 0.80 MgSO_4_, 1.01 NaH_2_PO_4_, 25 NaHCO_3_, 12 HEPES, 5.5 D-glucose, 1.8 CaCl_2_ and imaged live. Cells were then washed and incubated with MES acidic solution (pH 6.0) [sodium bicarbonate replaced in equimolar amount with membrane impermeable acid (MES)] and imaged again. Cells were then returned to the standard HEPES solution and a final live image was captured.
